# Wearing face masks regardless of symptoms is crucial for preventing the spread of COVID-19 in hospitals

**DOI:** 10.1017/ice.2020.202

**Published:** 2020-05-06

**Authors:** Joon Kee Lee, Hye Won Jeong

**Affiliations:** 1 Department of Pediatrics, Chungbuk National University Hospital, Cheongju, South Korea; 2 Department of Internal Medicine, Chungbuk National University College of Medicine, Cheongju, South Korea; 3 Department of Infection Control and Prevention, Chungbuk National University Hospital, Cheongju, South Korea

*To the Editor*—As of April 16, 2020, the number of confirmed cases of pandemic coronavirus disease 2019 (COVID-19) has reached 1,991,512, with 130,885 associated deaths.^1^ Although the numbers of confirmed cases and deaths continue to increase steeply through person-to-person transmission, asymptomatic or presymptomatic COVID-19 infections mean that mitigating community spread by isolating patients has limitations.^2,3^ Preventing outbreaks in healthcare centers is crucial because the demand for healthcare services is high, and mixing infected persons with those who are immunocompromised and/or elderly is almost unavoidable in these settings. The Centers for Disease Control and Prevention (CDC) of the United States and the Korean Centers for Diseases Control and Prevention (KCDC) have provided guidelines for infection control measures at healthcare facilities.^4,5^ In addition to the use of personal protective equipment by healthcare workers (HCWs), ensuring that all visiting patients and guardians wear face masks and adhere to strict hand hygiene protocols is crucial. Here, we share our experience in preventing the spread of SARS-CoV-2 within a hospital through strict monitoring at the hospital entrance by ensuring that all visitors wear face masks and practice strict hand hygiene.

In South Korea, the first COVID-19 patient was diagnosed on January 20, 2020. Since then, Chungbuk National University Hospital (CBNUH), an 810-bed referral hospital in Cheongju with ~2,300 employees and 3,000 outpatient visits per day, has undertaken hospital entrance control measures. These measures include reducing the number of unnecessary access points, checking the body temperatures of visitors using a thermal camera, and ensuring that all visitors and employees adhere to hand hygiene protocols and wear face masks, regardless of symptoms. The number of gates to the hospital was reduced from 5 to 2 during the day and to 1 at night.

On March 26, 2020, a point when almost 50% of COVID-19 cases in South Korea were imported from foreign countries, the regional public health office notified us that a 60-year-old female COVID-19 patient had visited CBNUH the day before. The KCDC requested an in-hospital epidemiologic investigation of all close contacts because they needed to quarantine them for at least 14 days. We started contact investigations using a photo of the patient and closed-circuit television recordings of the hospital entrance area. We identified the patient trying to enter the hospital. The alarm of the thermal camera was activated because the patient had a fever. To confirm the fever, a contactless thermometer was used by the hospital guard to check her body temperature, which was 38.3°C. The patient was guided to the COVID-19 screening clinic in a separate area of the emergency department. Nevertheless, the patient tried to re-enter the hospital through another gate. Similarly, the thermal camera was activated, and officials returned her to the COVID-19 screening clinic. The patient and all personnel who encountered her were wearing face masks. We identified 3 persons who had come into close contact with the patient: 2 hospital security guards who were wearing face masks, and 1 hospitalized patient who passed by within 2 meters and was not wearing a face mask. Even though the contact was brief, those who had close contact with this patient self-monitored with delegated supervision for 14 days,^6^ and none became ill with COVID-19.

Although face masks cannot completely prevent COVID-19, patients spread the virus through coughing,^7^ and face masks may reduce the number and travel distance of respiratory droplets.^8^ To prevent hospital spread of COVID-19, hospital entrance control, wearing of face masks, and strict hand hygiene protocols appear to be effective. Wearing eye shields protects HCWs in case of an accidental encounter with patients not wearing face masks.^4,5^ Individual preparedness in accord with major guidelines is crucial in preventing the spread of SARS-CoV-2 in healthcare centers.
